# Perioperative Glucagon-Like Peptide-1 Receptor Agonist Therapy and Postoperative Outcomes in Adult Foot and Ankle Surgery: A Scoping Review

**DOI:** 10.7759/cureus.101653

**Published:** 2026-01-15

**Authors:** Mohamed Zahed, Alzahraa Faris Alesawy, Ahmed Elkohail, Ahmed Ghazi, Adam Fell, Mohamed Hashem

**Affiliations:** 1 Orthopaedics, John Radcliffe Hospital, Oxford University Hospitals NHS Trust, Oxford, GBR; 2 Clinical Microbiology and Immunology, Faculty of Medicine, Benha University, Qalubiya, EGY; 3 Trauma and Orthopaedics, Princess Royal University Hospital, King's College NHS Trust, London, GBR; 4 Trauma and Orthopaedics, Warwick Hospital, Warwick, GBR; 5 Trauma and Orthopaedics, Imperial Healthcare Foundation Trust, London, GBR; 6 Orthopaedics, Frimley Health NHS Foundation Trust, London, GBR

**Keywords:** diabetes mellitus, foot and ankle surgery, fracture fixation, glp-1ra, non-union, orthopaedics surgery, pseudoarthrosis

## Abstract

Glucagon-like peptide-1 receptor agonists (GLP-1RAs) are emerging as a cornerstone in the management of type 2 diabetes and obesity. The perioperative implications of GLP-1RAs in orthopaedic surgery are a growing area of interest; however, their effects in patients undergoing foot and ankle surgery remain uncertain. This scoping review aims to map the available evidence on the use of GLP-1RAs in adults undergoing foot and/or ankle surgical procedures.

We aim to examine the impact of perioperative GLP-1RA use on postoperative outcomes in adults undergoing foot and/or ankle surgery.

We systematically searched PubMed, Embase, the Cochrane Library, Web of Science, and Scopus from January 2000 to July 2025. We included English-language full-text studies reporting on adult patients receiving GLP-1RAs who underwent foot and/or ankle surgery. Primary outcomes were bone union and early hardware removal after ankle fracture fixation. Secondary outcomes included infection, wound complications, reoperation, and post-traumatic arthritis. This scoping review was conducted in accordance with the PRISMA-ScR guidelines and the Joanna Briggs Institute (JBI) framework for scoping reviews.

A total of 102 records were identified, of which 88 remained after duplicate removal and underwent title and abstract screening. Three articles proceeded to full-text review; one was excluded because it investigated semaglutide in patients with chronic ankle instability without surgical intervention. Two retrospective, propensity-matched cohort studies using the TriNetX federated database met the inclusion criteria. Adults with diabetes undergoing tibiotalar, subtalar, or triple arthrodesis were evaluated and a lower overall rate of postoperative pseudarthrosis was reported among GLP-1RA users compared with matched controls (15.9% vs 20.2%; p = 0.0129). Subgroup analyses showed lower pseudarthrosis rates after subtalar fusion and triple arthrodesis in GLP-1RA users, with no significant difference after isolated tibiotalar fusion. Adults undergoing open reduction and internal fixation (ORIF) of ankle fractures were examined, and patients not receiving GLP-1RAs had a significantly higher 30-day hardware removal rate compared with GLP-1RA users (odds ratio 1.953; 95% confidence interval 1.062-3.591; p = 0.028). Neither study demonstrated an increased risk of postoperative infection or long-term complications in GLP-1RA users.

Current literature suggests that perioperative GLP-1RA use is associated with lower rates of pseudarthrosis after hindfoot arthrodesis and a reduced need for early hardware removal after ankle fracture fixation, without an apparent increase in postoperative infection or long-term complications. However, the evidence is limited, heterogeneous, and based solely on two retrospective database analyses with incomplete perioperative detail and potential residual confounding. This scoping review highlights a substantial gap in the literature regarding the use of GLP-1RAs in foot and ankle surgery and underscores the need for robust prospective studies to clarify the perioperative safety and efficacy of GLP-1RAs in adults undergoing foot and ankle surgery.

## Introduction and background

Glucagon-like peptide-1 receptor agonists (GLP-1RAs) have increasingly been recognised as promising therapeutic agents in the management of type 2 diabetes mellitus (T2DM) and obesity, owing to their potential for glycaemic control, weight loss, and cardiovascular benefits [1-4]. Although initially developed for the treatment of T2DM, large clinical trials have demonstrated their effectiveness in optimising metabolic parameters and body weight in patients with obesity [5,6]. Besides their well-known metabolic effects, GLP-1RAs may also have pleiotropic actions, such as influencing bone metabolism, aiding tissue repair, and modulating inflammation [7,8]. These properties raise essential questions about the effects of GLP-1RAs during and after surgical procedures, particularly when bone and soft tissue healing are critical.

In recent years, the use of GLP-1RA has grown quickly, with drugs like liraglutide, semaglutide, dulaglutide, exenatide, and tirzepatide being commonly prescribed to various patient groups [9]. GLP-1, an incretin hormone produced by enteroendocrine L-cells in the distal small intestine, is released when nutrients enter the gut. Its main effects are stimulating glucose-dependent insulin secretion, reducing glucagon release, slowing gastric emptying, and increasing feelings of fullness [10].

At the skeletal level, GLP-1 has been shown to influence bone metabolism by enhancing osteoblast differentiation and bone formation while reducing osteoclast-mediated bone resorption [11]. GLP-1 receptor activation may also support bone and soft tissue healing by improving microvascular perfusion and exerting anti-inflammatory effects [7,8]. Collectively, these pathways suggest that GLP-1RAs could modify postoperative outcomes in orthopaedic surgery.

Although clinical data remain limited, preoperative GLP-1RA use in arthroplasty studies has been associated with reduced periprosthetic joint infection rates and lower short-term readmission rates [12]. In the uniquely demanding surgical field of foot and ankle surgery, systemic comorbidities such as diabetes, obesity, and peripheral vascular disease may further complicate postoperative recovery [13,14]. Patients undergoing procedures such as arthrodesis or fracture fixation are at risk of complications, including delayed or non-union and hardware-related problems [14]. Given the ability of GLP-1RAs to improve glucose homeostasis, reduce body weight, and potentially modulate bone turnover, their perioperative effects on healing and complication rates have become a growing clinical concern [8].

During the perioperative period, anaesthetic concerns have arisen regarding delayed gastric emptying and aspiration risk in patients receiving GLP-1RAs [15,16]. Another concern involves identifying patient phenotypes most likely to benefit from perioperative GLP-1RA therapy or those at higher risk for complications. Modern diabetes management acknowledges that responses to GLP-1RA therapy vary among patients; factors like baseline glycaemic control, obesity level, severe peripheral neuropathy, and vascular disease burden likely influence how GLP-1RA exposure affects surgical outcomes [17,18]. While guidance has started to emerge on how to manage these medications around the time of surgery, less is known about their longer-term effects on postoperative outcomes such as infection, wound healing, and osseous union. These knowledge gaps are particularly relevant to orthopaedic surgeons and perioperative physicians.

The current evidence base addressing the postoperative impact of GLP-1RAs in foot and ankle surgery is notably sparse, despite the heightened mechanical stresses inherent to healing in this anatomical region. Particular attention should be paid to the relationship between GLP-1RAs and foot and ankle surgery, given the complex interplay of systemic disease, impaired mobility, and local biomechanical challenges in this patient group [13]. Through this scoping review, we aim to map and characterise all available studies assessing the postoperative effects of GLP-1RAs in adults undergoing foot and ankle operations.

## Review

Methods

Study Design

We conducted this scoping review in line with the Joanna Briggs Institute (JBI) methodological approach [19,20], and adhered to the PRISMA-ScR checklist to ensure transparent and comprehensive reporting [21]. The JBI Population-Concept-Context (PCC) framework is provided in Table 1 [19-21]. The protocol for this review was submitted and prospectively registered in the PROSPERO database (ID: CRD420251178612).

**Table 1:** Inclusion and exclusion criteria based on the Population-Concept-Context (PCC) framework adapted from the Joanna Briggs Institute (JBI) methodology.

Term	Inclusion Criteria	Exclusion Criteria
Population	• Adults (≥18 years) undergoing foot and/or ankle surgery of any type, including trauma (e.g., fracture fixation), elective (e.g., arthrodesis, deformity correction), or soft-tissue procedures. • Patients with or without type 2 diabetes or obesity.	• Patients under 18 years of age. • Non-human/animal studies. • Studies that do not specify foot or ankle surgical procedures (e.g., general orthopaedic or non-surgical populations).
Concept	• Studies evaluating glucagon-like peptide-1 receptor agonist (GLP-1RA) exposure (e.g., semaglutide, liraglutide, dulaglutide, exenatide, lixisenatide, tirzepatide, albiglutide). • Studies reporting postoperative outcomes such as pseudarthrosis, infection, wound healing, hardware removal, non-union, or other complications. • All study designs (RCTs, cohort, case-control, case series, registry analyses).	• Studies without any GLP-1RA exposure data. • Studies focusing solely on biochemical, pharmacokinetic, or non-surgical effects of GLP-1RAs. • Studies assessing GLP-1RAs in non-orthopaedic or non-surgical contexts.
Context	• Studies conducted in hospital or surgical settings (inpatient, outpatient, or ambulatory). • Any country or healthcare system. • Peer-reviewed primary research, secondary data analyses, or relevant reviews providing extractable postoperative outcomes.	• Editorials, letters, protocols, commentaries, and non-peer-reviewed content. • Studies with no extractable data related to foot or ankle surgery outcomes. • Non-English publications.

Search Strategy

We conducted a comprehensive literature search across five databases: PubMed, Web of Science, Scopus, Embase, and the Cochrane Library, covering the period from January 2000 to July 2025. To maximise completeness, we conducted a manual search of the reference lists of relevant publications to locate any further studies suitable for inclusion. Our search approach utilised a combination of controlled indexing terms and free-text keywords pertaining to GLP-1RA therapy and foot or ankle surgical procedures. Table 2 contains the full search strategies for all databases.

**Table 2:** The complete search strategies for each database

Search Terms and Strategy Utilised	Database	Studies Retrieved
("Glucagon-Like Peptide 1" OR "GLP-1" OR "GLP1" OR "glucagon like peptide-1" OR "semaglutide" OR "liraglutide" OR "dulaglutide" OR "exenatide" OR "albiglutide") AND ("Foot Surgery" OR "Ankle Surgery" OR "Foot and Ankle" OR "Orthopaedic Surgery" OR "Podiatric Surgery" OR "Lower Extremity Surgery" OR "Foot Injuries/surgery"[MeSH] OR "Ankle Injuries/surgery"[MeSH])	PubMed	51
('glucagon like peptide 1 agonist' OR 'glucagon like peptide 1' OR 'glp 1' OR 'glp1' OR semaglutide OR liraglutide OR dulaglutide OR exenatide OR albiglutide) AND ('foot surgery' OR 'ankle surgery' OR 'podiatric surgery' OR 'lower extremity surgery' OR 'foot and ankle surgery' OR 'orthopedic surgery' OR 'orthopaedic surgery')	Embase	20
("glucagon like peptide 1" OR "glp-1" OR "glp1" OR semaglutide OR liraglutide OR dulaglutide OR exenatide OR albiglutide) AND ("foot surgery" OR "ankle surgery" OR "foot and ankle surgery" OR "podiatric surgery" OR "lower limb surgery" OR "orthopedic surgery" OR "orthopaedic surgery")	Cochrane	1
( "Glucagon-Like Peptide 1" OR "GLP-1" OR "GLP1" OR "glucagon like peptide-1" OR "semaglutide" OR "liraglutide" OR "dulaglutide" OR "exenatide" OR "albiglutide" ) AND ( "Foot Surgery" OR "Ankle Surgery" OR "Foot and Ankle" OR "Orthopedic Procedures" OR "Orthopaedic Surgery" OR "Podiatric Surgery" OR "Lower Extremity Surgery" OR "Foot Injuries/surgery" [MeSH] OR "Ankle Injuries/surgery" [MeSH] )	Web of Science	7
( "Glucagon-Like Peptide 1" OR "GLP-1" OR "GLP1" OR "glucagon like peptide-1" OR "semaglutide" OR "liraglutide" OR "dulaglutide" OR "exenatide" OR "albiglutide" ) AND ( "Foot Surgery" OR "Ankle Surgery" OR "Foot and Ankle" OR "Orthopedic Procedures" OR "Orthopaedic Surgery" OR "Podiatric Surgery" OR "Lower Extremity Surgery" OR "Foot Injuries/surgery" [MeSH] OR "Ankle Injuries/surgery" [MeSH] )	Scopus	23
Citation tracking performed within relevant papers.		0

Inclusion and Exclusion Criteria

Using the Population-Concept-Context framework, we defined our eligibility criteria as follows. The population comprised adults aged 18 years and above who had foot and/or ankle operations, spanning trauma-related procedures such as fracture fixation and elective surgeries like arthrodesis or deformity correction. The review concept encompassed perioperative administration of GLP-1 receptor agonists including semaglutide, liraglutide, dulaglutide, exenatide, lixisenatide, tirzepatide, and albiglutide and their associations with postoperative outcomes such as infection, pseudarthrosis, delayed wound healing, non-union, subsequent hardware removal or other complications. The context was hospital or surgical settings (inpatient, outpatient, or ambulatory) across any healthcare system and country.

We included English-language publications encompassing randomised controlled trials, cohort and case-control designs, in addition to case series and case reports. Studies were excluded if they were not published in English, were editorials or commentaries, appeared only as conference abstracts, involved animal or bench research, or did not provide postoperative data specific to foot and ankle procedures.

Study Selection

**Figure 1:**
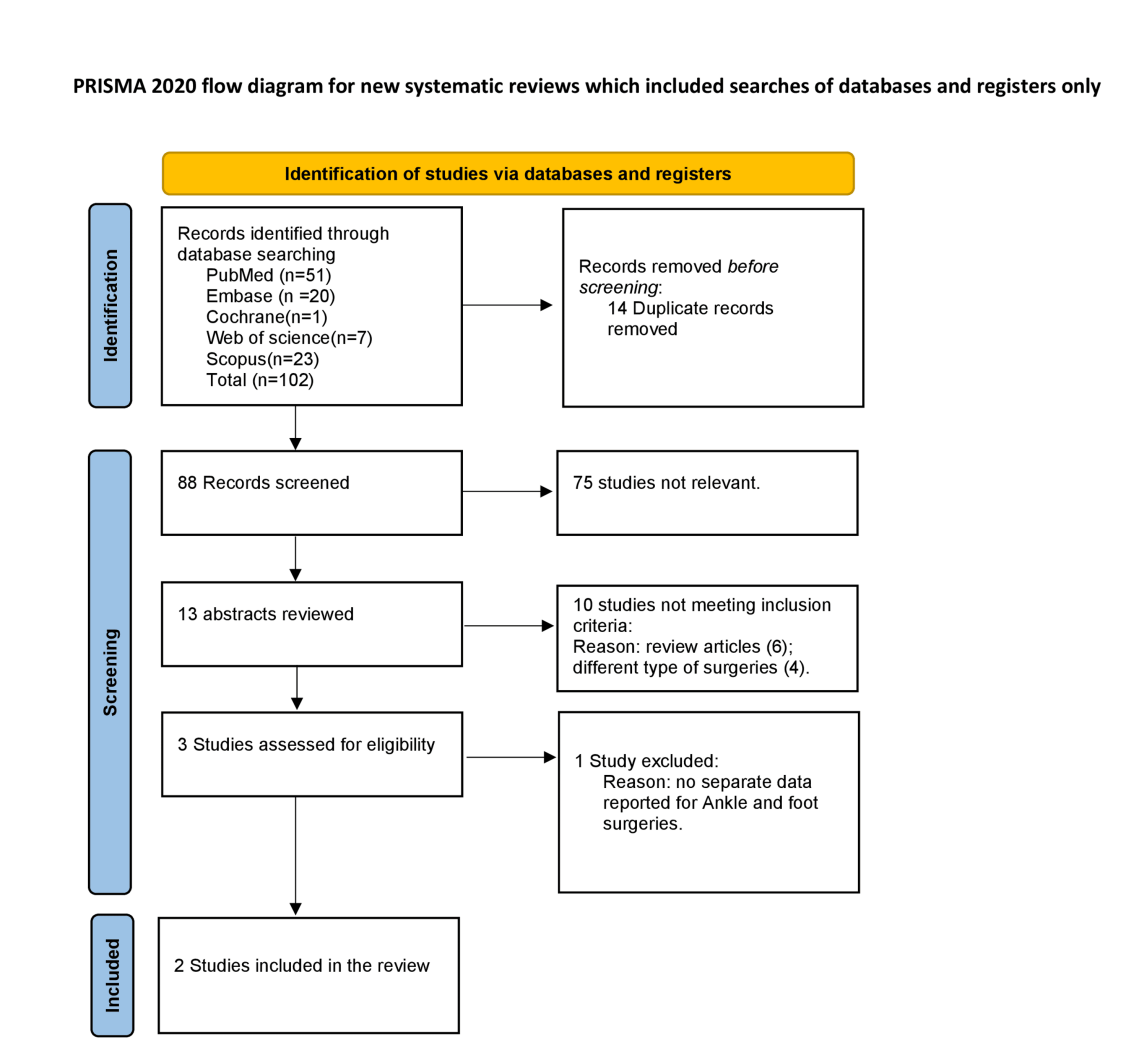
The PRISMA-ScR flow diagram illustrating the study selection process

All retrieved records were imported into Rayyan, a web-based reference management and screening tool, for duplicate removal. The screening process involved an initial assessment of titles and abstracts, with eligible records undergoing subsequent full-text review. Two independent authors screened all records, and a third reviewer resolved disagreements. A total of 102 records were initially identified. After removal of duplicates, 88 unique records underwent title and abstract screening. Three articles proceeded to full-text review. One study, which assessed semaglutide use in patients with chronic ankle instability without surgical intervention, was excluded. Two studies met the inclusion criteria and were ultimately included in the scoping review. The PRISMA-ScR flow diagram illustrating the study selection process is presented in Figure 1.

Data Charting and Extraction

Two independent reviewers used a standardised data extraction form to ensure consistency and accuracy across included studies. Extracted data included bibliographic details (study identifier, authors, study design, year of publication, and country); participant and surgical characteristics (sample size, demographics, comorbidities, and procedure type); GLP-1RA exposure (drug type, exposure window, and dosing information where available); and postoperative outcomes with key findings (infection, pseudarthrosis or non-union, wound complications, reoperation, hardware removal, post-traumatic arthritis, and other reported complications).

Characteristics of the included studies are summarised in Table 3 [21-23].

**Table 3:** Data extraction sheet for included studies EHR: Electronic health record

Study ID	Study design	Data range (in years)	Population characteristics	Study groups	Type of medication	Outcomes	Limitations
Levidy et al., 2025 [22]	Retrospective cohort study	2005-2024	Type 2 diabetes mellitus -tibiotalar fusion, subtalar fusion, and triple arthrodesis	GLP-1 and without GLP-1	Tirzepatide, Semaglutide, lixisenatide, dulaglutide, liraglutide, and pramlintide.	GLP-1 agonist users demonstrated a lower overall rate of postoperative pseudarthrosis following ankle arthrodesis. This benefit was specifically observed in patients undergoing subtalar fusion and triple arthrodesis, while tibiotalar fusion showed no significant difference between groups. Postoperative infection rates remained comparable regardless of GLP-1 agonist use across all procedure types [20].	This retrospective study is limited by coding accuracy, especially for tibiotalar fusions, where cross-coding may increase outcome variability. It couldn't evaluate individual imaging, functional status, or fusion quality indicators like refracture [20]. Diagnostic criteria for pseudarthrosis varied among providers—some based on clinical assessment, others on radiography or CT scans. This inconsistency is notable considering GLP-1 agonists' potential to reduce bone remodelling by decreasing osteoclast activity [20]. Conflicting indications for GLP-1 use complicate interpretation, as the study covers the 2015 FDA approval; before that, these agents were only for diabetes, with different dosages. Therapy duration may also confound results since bone effects may need longer exposure than the rapid glycemic effects seen within days. Prospective trials are needed to clarify GLP-1 agonists' impact on bone healing [20].
Morningstar et al., 2025 [23]	Retrospective cohort study	2000-2024	Patients aged 18 and above- operative fixation of an ankle fracture.	GLP-1 and without GLP-1	Tirzepatide, Liraglutide, Exenatide, Pramlintide, Dulaglutide, and Semaglutide	At 30 days, non-GLP-1RA patients had significantly higher rates of ankle ORIF hardware removal compared to GLP-1RA users (OR = 1.953; 95% CI = 1.062–3.591; p = 0.028), with no other complications showing significant differences. At 90 days, 1 year, and 5 years of follow-up, no significant differences in complication risk were observed between groups [21]. Regarding post-traumatic arthritis following ankle fracture ORIF, GLP-1RA use was not associated with significant differences in development rates within 5 years (OR = 1.313; 95% CI = 0.895–1.927) or at any time after surgery (OR = 1.323; 95% CI = 0.909–1.925) [21].	This retrospective EHR analysis relies on accurate coding, which is susceptible to errors. Outcomes were identified using ICD-10-CM codes rather than radiographic images or provider notes, potentially leading to inaccurate counts of adverse outcomes [21]. To reduce confounding, 1:1 propensity score matching was performed based on demographics and comorbidities linked to adverse surgical outcomes. However, GLP-1RA patients were identified by prescriptions within 1 year of surgery, but medication compliance, dosing, and frequency couldn't be verified. More high-level studies considering additional diabetic and obesity factors are needed [21].

Risk of Bias Assessment

In line with JBI guidance for scoping reviews, no formal risk-of-bias assessment was undertaken [19,20]. Instead, we conducted a narrative appraisal of methodological limitations, focusing on study design, data source, propensity score-matching methodology, and the risk of residual confounding.

Synthesis of Results

Given the limited number of included studies, the immaturity of this research area, and the heterogeneity of reported outcomes, data were summarised narratively. No quantitative synthesis or meta-analysis was conducted.

Results

Two studies met the inclusion criteria: Levidy et al. (2025) [22] and Morningstar et al. (2025) [23]. Both were retrospective, propensity-matched cohort analyses using the TriNetX federated electronic health record network. Levidy et al. evaluated adults with T2DM undergoing tibiotalar, subtalar, or triple arthrodesis. Patients were divided into two cohorts: GLP-1RA users and a control group without GLP-1RA exposure. Outcomes were assessed at one year postoperatively and included pseudarthrosis (non-union) and postoperative infection [22]. Morningstar et al. included adult patients who underwent ORIF of ankle fractures between 2000 and 2024 [23]. Two cohorts were established according to preoperative GLP-1RA use, and 1:1 propensity score matching was used to create balanced groups [23]. Primary outcomes were postoperative complications at 30 days, 90 days, 1 year, and 5 years, including infection, non-union, reoperation, and hardware removal [23].

Levidy et al. compared two cohorts of 783 patients with T2DM undergoing tibiotalar, subtalar, or triple arthrodesis: those treated with GLP-1RAs and matched controls without GLP-1RA use [22]. Among GLP-1RA users, the overall rate of postoperative pseudarthrosis was lower than in the control cohort (15.9% vs 20.2%; p = 0.0129). When analysed by procedure type, GLP-1RA use was associated with lower pseudarthrosis rates after subtalar fusion (17.2% vs 23.4%; p = 0.0292) and triple arthrodesis (12.4% vs 21.9%; p = 0.0120) [22]. No significant difference in pseudarthrosis was observed after tibiotalar fusion (19.8% vs 21.5%; p = 0.5692). No differences in postoperative infection rates were detected between GLP-1RA users and controls across any procedure type [22].

Morningstar et al. studied 123,546 patients not taking GLP-1RAs and 1,173 patients taking GLP-1RAs who underwent ORIF for ankle fractures, with 1:1 propensity score matching resulting in two cohorts of 1,173 patients each [23]. Following matching, no significant differences were found in demographics or comorbidities, including the rates of T2DM and obesity [23].

At 30 days post-surgery, the group not receiving GLP-1RAs had a notably higher rate of hardware removal compared to the GLP-1RA group (odds ratio 1.953; 95% confidence interval 1.062-3.591; p = 0.028) [21]. Other complications did not differ significantly at this time point. At 90 days, 1 year, and 5 years postoperatively, there were no significant differences in complications, including the occurrence of post-traumatic ankle arthritis. The authors concluded that preoperative GLP-1RA use did not increase complication rates and may decrease the need for early hardware removal following ankle fracture ORIF [23].

Discussion

This scoping review aimed to characterise the potential associations between GLP-1RA exposure and postoperative outcomes in adult foot and ankle surgery, with emphasis on bone-healing measures and complications such as infection and hardware failure. Despite the rapidly increasing use of GLP-1RAs for T2DM and obesity, we identified only two eligible studies addressing postoperative outcomes in this specific surgical population. Both were retrospective database studies using the TriNetX network [22,23].

Within these limitations, the available evidence suggests that GLP-1RA use is not associated with an increased risk of postoperative complications or infection in adult foot and ankle surgery. Interestingly, both studies described potential favourable associations. Levidy et al. reported that patients on GLP-1RAs experienced lower pseudarthrosis rates after subtalar and triple arthrodesis [22], while Morningstar et al. reported a lower rate of early hardware removal after ankle fracture ORIF in patients receiving GLP-1RAs [23].

These findings are broadly consistent with emerging evidence from larger arthroplasty cohorts. Lee et al. reported improved early surgical outcomes and reduced 90-day readmission rates in GLP-1RA-treated patients undergoing total hip and knee arthroplasty [24], while Buddhiraju et al. found a decreased risk of periprosthetic joint infection and lower readmission rates among diabetic patients on GLP-1RAs following joint arthroplasty [12]. Conversely, Heo et al. observed no significant differences in complication or infection rates between GLP-1RA-treated and non-treated patients undergoing total knee arthroplasty [25]. Overall, these data support the perioperative safety of GLP-1RAs and suggest possible orthopaedic benefits.

From a mechanistic perspective, GLP-1RAs may exert favourable effects on bone and soft tissue healing through improved glycaemic control, weight reduction, anti-inflammatory actions, and enhanced microvascular perfusion. By improving glycaemic regulation and reducing immune dysfunction associated with obesity, GLP-1RA therapy may indirectly decrease the likelihood of postoperative infection; at the same time, its effects on bone metabolism could enhance both fusion success and fracture healing [7,8,11,26].

Notably, experimental studies have shown that GLP-1 enhances osteoblast activity, upregulates bone morphogenetic signalling, and improves microvascular perfusion. The high risk of pseudarthrosis in hindfoot fusion procedures may make these pathways particularly crucial [27]. Furthermore, the weight reduction achieved with GLP-1RA therapy may decrease loading across the subtalar and ankle joints, potentially enhancing the biomechanical environment necessary for successful fusion [8]. Yet none of the available clinical studies report medication timing, adherence, dosing, or perioperative weight trajectories, making it impossible to distinguish metabolic effects from confounding behavioural or physiological factors. Besides that, the high frequency of neuropathy, microvascular disease, and chronic swelling in foot and ankle patients highlights the complex interactions among GLP-1RA use, tissue perfusion, and mechanical stress [28].

Clinical and Research Prospects

From a clinical perspective, the potential influence of GLP-1RAs on postoperative healing has essential implications for foot and ankle surgeons, who often manage patients with complex metabolic and vascular comorbidities [29]. Clinically, our findings are encouraging. In a patient population frequently burdened by diabetes, obesity, and vascular compromise, it is reassuring that GLP-1RA use has not been associated with higher postoperative infection rates and may be linked to improved union and fewer hardware-related complications in selected contexts.

For surgeons, this raises the possibility that GLP-1RAs may serve as an adjunctive factor in optimising metabolic state before procedures with high mechanical and biological demands, such as ankle fracture fixation or hindfoot arthrodesis.

At present, however, practical decision-making remains challenging due to anaesthetic concerns about delayed gastric emptying and inconsistent institutional protocols [30]. Moreover, the varied occurrence of diabetic complications in foot and ankle patients - including neuropathy in up to 50% of those with advanced peripheral vascular disease - indicates that GLP-1RA benefits for bone healing and infection prevention are likely greatest in patients who are well-controlled and metabolically stable. Conversely, individuals with severe neuropathy or significant vascular impairment may experience limited benefits or potential risks from medication-induced alterations in sensory perception or vascular tone [16,18]. Until robust prospective evidence becomes available, clinicians should adopt an individualised approach that considers glycaemic control, obesity, aspiration risk, and the specific surgical procedure, while maintaining close communication between surgical, anaesthetic, and diabetes teams to guide perioperative medication planning.

Future research should focus on well-designed prospective cohort studies and, where feasible, randomised controlled trials that incorporate detailed data on GLP-1RA regimens (dose, duration, timing of initiation and cessation), patient-level metabolic control (e.g., HbA1c trajectories), body mass index, smoking status, nutritional status, and concomitant medications. Subgroup analyses stratified by diabetic control, obesity severity, and procedure type (e.g., ankle fusion vs subtalar fusion vs ankle fracture ORIF) will be essential to clarify which patients derive the most significant benefit and whether any high-risk subgroups exist.

Strengths and Limitations

This review has several strengths. It addresses an under-recognised clinical question through a comprehensive, up-to-date literature search across multiple databases. The use of both the JBI methodological framework and PRISMA-ScR reporting standards strengthens the transparency and reproducibility of the review. Independent screening by multiple reviewers was employed to minimise selection bias and improve reliability. This is the first review specifically examining the impact of GLP-1RAs on postoperative outcomes in adult foot and ankle surgery.

However, significant limitations must be acknowledged. First, the evidence base is minimal, with only two eligible studies and no prospective clinical trials. Although these studies encompassed substantial patient numbers, their retrospective design and reliance on a single federated data source (TriNetX) introduce potential selection bias and residual confounding. Second, key variables such as precise medication timing, adherence, perioperative adjustments, and detailed glycaemic or weight trajectories were not available. Third, although both studies used propensity score matching, unmeasured confounders (e.g., frailty, nutritional status, detailed vascular assessment) may still influence the observed associations. Finally, restricting inclusion to English-language publications may have excluded relevant work in other languages.

## Conclusions

Current literature suggests that perioperative GLP-1RA use is associated with lower rates of pseudarthrosis after hindfoot arthrodesis and a reduced need for early hardware removal after ankle fracture fixation, without an apparent increase in postoperative infection or long-term complications. However, the evidence is limited, heterogeneous, and based solely on two retrospective database analyses with incomplete perioperative detail and potential residual confounding. This scoping review highlights a substantial gap in the literature regarding the use of GLP-1RAs in foot and ankle surgery. It underscores the need for robust prospective studies to clarify their perioperative safety and efficacy in adults undergoing foot and ankle surgery.
